# Supplementary material for: Sex-based differences in long-term outcomes after stroke: A meta-analysis
Source: PLoS One. 2023 Apr 27;18(4):e0283204. doi: 10.1371/journal.pone.0283204 (PMC10138847; doi:10.1371/journal.pone.0283204)
Supplement: S1 File — (PDF) [file pone.0283204.s002.pdf]

SUPPLEMENTAL MATERIAL

Sex difference in long term outcomes after stroke: A Meta-Analysis

Detail search query

Supplement figure Forest plot for 1-year mortality, stroke recurrence and favorable outcome.

Detailed search query

1. PubMed

(Final search run on 17-08-2022; 480 items)

((((((((((((((sex[MeSH Terms]) OR (gender[Title/Abstract])) OR ('Sex Difference*'[Title/Abstract])) OR ('Gender Difference*'[Title/Abstract])) OR ('Sex Characteristic*'[Title/Abstract])) OR (Women[MeSH Terms])) OR (Woman[Title/Abstract])) OR (Female[Title/Abstract])) OR (men[MeSH Terms]))) OR (man[Title/Abstract])) OR (Female[Title/Abstract])) AND (long term[Title/Abstract])) AND (((outcome[MeSH Terms]) OR (outcome[Title/Abstract])) OR (outcomes[Title/Abstract]))) AND ((((((((((stroke[MeSH Terms]) OR (strokes[Title/Abstract])) OR (Cerebrovascular Accident[Title/Abstract])) OR (Cerebrovascular Accidents[Title/Abstract])) OR (CVA (Cerebrovascular Accident[Title/Abstract]))) OR (CVAs (Cerebrovascular Accident[Title/Abstract]))) OR (Cerebral Stroke[Title/Abstract])) OR (Cerebral Strokes[Title/Abstract])) OR (Stroke, Cerebral[Title/Abstract])) OR (Acute Stroke[Title/Abstract]))

2. Embase

(Final search run on 17-08-2022; 1095 items)

((((((((((((((sex: ti,ab.) OR (gender: ti.ab.)) OR ('Sex Difference*': ti.ab.)) OR ('Gender Difference*': ti.ab.)) OR ('Sex Characteristic*': ti.ab.)) OR (Women: ti.ab.)) OR (Woman: ti.ab.)) OR (Female: ti.ab.)) OR (men: ti.ab.))) OR (man: ti.ab.)) OR (Female: ti.ab.)) AND (long term: ti.ab.)) AND (((outcome: ti.ab.) OR (outcome: ti.ab.)) OR (outcomes: ti.ab.))) AND ((((((((((stroke: ti.ab.) OR (strokes: ti.ab.)) OR (Cerebrovascular Accident: ti.ab.)) OR (Cerebrovascular Accidents: ti.ab.)) OR (CVA (Cerebrovascular Accident: ti.ab.))) OR (CVAs (Cerebrovascular Accident: ti.ab.))) OR (Cerebral Stroke: ti.ab.)) OR (Cerebral Strokes: ti.ab.)) OR (Stroke, Cerebral: ti.ab.)) OR (Acute Stroke: ti.ab.))

3. Cochrane

(Final search run on 17-08-2022; 57 items)

((((((((((((((sex[MeSH Terms]) OR (gender[Title/Abstract])) OR ('Sex Difference*'[Title/Abstract])) OR ('Gender Difference*'[Title/Abstract])) OR ('Sex Characteristic*'[Title/Abstract])) OR (Women[MeSH Terms])) OR (Woman[Title/Abstract])) OR (Female[Title/Abstract])) OR (men[MeSH Terms]))) OR (man[Title/Abstract])) OR (Female[Title/Abstract])) AND (long term[Title/Abstract])) AND (((outcome[MeSH Terms]) OR (outcome[Title/Abstract])) OR (outcomes[Title/Abstract]))) AND ((((((((((stroke[MeSH Terms]) OR (strokes[Title/Abstract])) OR (Cerebrovascular Accident[Title/Abstract])) OR (Cerebrovascular Accidents[Title/Abstract])) OR (CVA (Cerebrovascular Accident[Title/Abstract]))) OR (CVAs (Cerebrovascular Accident[Title/Abstract]))) OR (Cerebral Stroke[Title/Abstract])) OR (Cerebral Strokes[Title/Abstract])) OR (Stroke, Cerebral[Title/Abstract])) OR (Acute Stroke[Title/Abstract]))

A. 1-year mortality

Study or Subgroup	male Events	male Total	female Events	female Total	Weight	Odds Ratio M-H, Random, 95% CI
Li 2015	107	488	58	383	11.1%	1.57 [1.11, 2.24]
Wang 2013	875	7118	789	4442	15.2%	0.65 [0.58, 0.72]
Sheikh 2007	2730	9364	3697	13251	15.6%	1.06 [1.00, 1.13]
Geng 2019	17	147	23	81	5.9%	0.33 [0.16, 0.66]
Wang 2022	198	6236	109	2802	13.2%	0.81 [0.64, 1.03]
Vemmos 2000	106	309	98	245	11.2%	0.78 [0.55, 1.11]
Kong 2010	177	1655	151	1119	13.3%	0.77 [0.61, 0.97]
Medlin 2020	409	2236	372	1757	14.5%	0.83 [0.71, 0.97]
**Total (95% CI)**		**27553**		**24080**	**100.0%**	**0.83 [0.67, 1.03]**
Total events	4619		5297			

Heterogeneity: $Tau^2 = 0.08$; $Chi^2 = 89.35$, df = 7 (P < 0.00001); $I^2 = 92\%$
Test for overall effect: Z = 1.71 (P = 0.09)

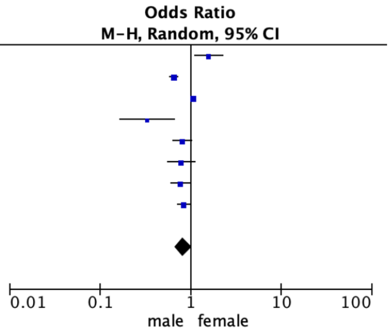

B. 1-year favorable outcome

Study or Subgroup	male Events	male Total	female Events	female Total	Weight	Odds Ratio M-H, Random, 95% CI
Wang 2022	5435	6236	2344	2802	60.5%	1.33 [1.17, 1.50]
Kong 2010	778	1655	432	1119	39.5%	1.41 [1.21, 1.65]
**Total (95% CI)**		**7891**		**3921**	**100.0%**	**1.36 [1.23, 1.50]**
Total events	6213		2776			

Heterogeneity: $Tau^2 = 0.00$; $Chi^2 = 0.38$, df = 1 (P = 0.54); $I^2 = 0\%$
Test for overall effect: Z = 6.20 (P < 0.00001)

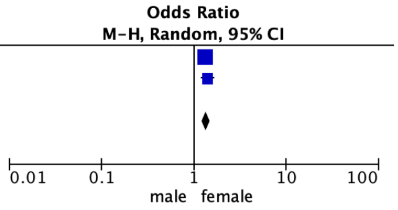

C. 1-year stroke recurrence

Study or Subgroup	male Events	male Total	female Events	female Total	Weight	Odds Ratio M-H, Random, 95% CI
Wang 2013	1167	7118	883	4442	42.2%	0.79 [0.72, 0.87]
Li 2015	164	488	114	383	21.1%	1.19 [0.90, 1.59]
Wang 2022	619	6236	315	2802	36.6%	0.87 [0.75, 1.00]
**Total (95% CI)**		**13842**		**7627**	**100.0%**	**0.89 [0.75, 1.07]**
Total events	1950		1312			

Heterogeneity: $Tau^2 = 0.02$; $Chi^2 = 7.46$, df = 2 (P = 0.02); $I^2 = 73\%$
Test for overall effect: Z = 1.25 (P = 0.21)

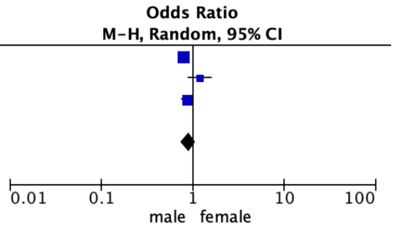

Supplement figure Forest plot for 1-year mortality, stroke recurrence and favorable outcome.
