# Supplementary material for: Sex-based differences in long-term outcomes after stroke: A meta-analysis
Source: PLoS One. 2023 Apr 27;18(4):e0283204. doi: 10.1371/journal.pone.0283204 (PMC10138847; doi:10.1371/journal.pone.0283204)
Supplement: Attachment — Submitted filename: PONE-D-23-00740_reviewer_IS.pdf [file pone.0283204.s003.pdf]

PLOS ONE

Sex-based differences in long-term outcomes after stroke: a meta-analysis

--Manuscript Draft--

**Manuscript Number:**	PONE-D-23-00740
**Article Type:**	Research Article
**Full Title:**	Sex-based differences in long-term outcomes after stroke: a meta-analysis
**Short Title:**	sex-based difference after stroke
**Corresponding Author:**	Feng Zheng, M.D.<br>Second Affiliated Hospital of Fujian Medical University<br>CHINA
**Keywords:**	Male, Female, Stroke, Long-term outcomes
**Abstract:**	Abstract<br>Background: There is limited data on sex-related disparities in the long-term outcomes after stroke. We aim to investigate whether there are sex-based differences in long-term outcomes using pooled data.<br>Methods: Three databases (PubMed, Embase, and Cochrane Library) were systematically searched from inception to July 2022. This meta-analysis was performed in accordance with the recommendations and guidelines of the Preferred Reporting Items for Systematic Reviews and Meta-Analyses. The modified Newcastle-Ottawa scale was used to assess the risk of bias. In addition, a random-effects model was used.<br>Results: Twenty-two cohort studies with 84538 patients were included. There were 50.2% men and 49.8% women. Women had a higher mortality at 1 (odds ration [OR], 0.82; 95% confidence interval [CI][0.69, 0.99], P=0.03) and 10 (OR 0.72, 95% CI[0.65, 0.79], P < 0.00001) years, higher stroke recurrence at 1 year (OR 0.85, 95% CI[0.73, 0.98], P=0.02), lower favorable outcome at 1 year (OR 1.36, 95% CI[1.24, 1.49], P < 0.00001). No significant difference was detected between men and women in the outcomes of health-related quality of life and depression.<br>Conclusion: In this meta-analysis, the mortality at 1- and 5-year and stroke recurrence rates were higher in female patients than in male patients after stroke. In addition, females tended to experience less favorable outcomes in the first year after stroke. Finally, further long-term studies on sex disparities in stroke prevention, care, and management are warranted to explore the opportunities to reduce this gap.
**Order of Authors:**	Xiumei Guo
	Xiong Yu
	Xinyue Huang
	Zhigang Pan
	Xiaodong Kang
	Chunhui Chen
	Jianfeng Zhou
	Hanlin Zheng
	Yuping Chen
	Weipeng Hu
	Lingxing Wang
	Feng Zheng, M.D.
**Additional Information:**	
**Question**	**Response**
**Financial Disclosure**	The author(s) received no specific funding for this work.

Powered by Editorial Manager® and ProduXion Manager® from Aries Systems Corporation

Enter a financial disclosure statement that describes the sources of funding for the work included in this submission. Review the submission guidelines for detailed requirements. View published research articles from *PLOS ONE* for specific examples.<br><br>This statement is required for submission and **will appear in the published article** if the submission is accepted. Please make sure it is accurate.<br><br>**Unfunded studies**<br>Enter: *The author(s) received no specific funding for this work.*<br><br>**Funded studies**<br>Enter a statement with the following details:<br>• Initials of the authors who received each award<br>• Grant numbers awarded to each author<br>• The full name of each funder<br>• URL of each funder website<br>• Did the sponsors or funders play any role in the study design, data collection and analysis, decision to publish, or preparation of the manuscript?<br>• **NO** - Include this sentence at the end of your statement: *The funders had no role in study design, data collection and analysis, decision to publish, or preparation of the manuscript.*<br>• **YES** - Specify the role(s) played.<br><br>* typeset	
**Competing Interests**<br><br>Use the instructions below to enter a competing interest statement for this submission. On behalf of all authors, disclose any competing interests that could be perceived to bias this work—acknowledging all financial support and any other relevant financial or non-financial competing interests.<br><br>This statement is required for submission and **will appear in the published article** if	The authors have declared that no competing interests exist.

the submission is accepted. Please make sure it is accurate and that any funding sources listed in your Funding Information later in the submission form are also declared in your Financial Disclosure statement.<br><br>View published research articles from *PLOS ONE* for specific examples.<br><br>**NO authors have competing interests**<br><br>Enter: *The authors have declared that no competing interests exist.*<br><br>**Authors with competing interests**<br><br>Enter competing interest details beginning with this statement:<br><br>*I have read the journal's policy and the authors of this manuscript have the following competing interests: [insert competing interests here]*<br><br>* typeset	
**Ethics Statement**<br><br>Enter an ethics statement for this submission. This statement is required if the study involved:<br><br>• Human participants<br>• Human specimens or tissue<br>• Vertebrate animals or cephalopods<br>• Vertebrate embryos or tissues<br>• Field research<br><br>Write "N/A" if the submission does not require an ethics statement.<br><br>General guidance is provided below. Consult the submission guidelines for detailed instructions. **Make sure that all information entered here is included in the Methods section of the manuscript.**	N/A

**Format for specific study types**<br><br>**Human Subject Research (involving human participants and/or tissue)**<br>• Give the name of the institutional review board or ethics committee that approved the study<br>• Include the approval number and/or a statement indicating approval of this research<br>• Indicate the form of consent obtained (written/oral) or the reason that consent was not obtained (e.g. the data were analyzed anonymously)<br><br>**Animal Research (involving vertebrate animals, embryos or tissues)**<br>• Provide the name of the Institutional Animal Care and Use Committee (IACUC) or other relevant ethics board that reviewed the study protocol, and indicate whether they approved this research or granted a formal waiver of ethical approval<br>• Include an approval number if one was obtained<br>• If the study involved *non-human primates*, add *additional details* about animal welfare and steps taken to ameliorate suffering<br>• If anesthesia, euthanasia, or any kind of animal sacrifice is part of the study, include briefly which substances and/or methods were applied<br><br>**Field Research**<br><br>Include the following details if this study involves the collection of plant, animal, or other materials from a natural setting:<br>• Field permit number<br>• Name of the institution or relevant body that granted permission	
**Data Availability**<br><br>Authors are required to make all data underlying the findings described fully available, without restriction, and from the time of publication. PLOS allows rare exceptions to address legal and ethical concerns. See the PLOS Data Policy and FAQ for detailed information.	Yes - all data are fully available without restriction

A Data Availability Statement describing where the data can be found is required at submission. Your answers to this question constitute the Data Availability Statement and **will be published in the article**, if accepted.<br><br>**Important:** Stating 'data available on request from the author' is not sufficient. If your data are only available upon request, select 'No' for the first question and explain your exceptional situation in the text box.<br><br>Do the authors confirm that all data underlying the findings described in their manuscript are fully available without restriction?	
**Describe where the data may be found in full sentences. If you are copying our sample text, replace any instances of XXX with the appropriate details.**<br><br>• If the data are **held or will be held in a public repository**, include URLs, accession numbers or DOIs. If this information will only be available after acceptance, indicate this by ticking the box below. For example: *All XXX files are available from the XXX database (accession number(s) XXX, XXX.*).<br>• If the data are all contained **within the manuscript and/or Supporting Information files**, enter the following: *All relevant data are within the manuscript and its Supporting Information files.*<br>• If neither of these applies but you are able to provide **details of access elsewhere**, with or without limitations, please do so. For example:<br><br>*Data cannot be shared publicly because of [XXX]. Data are available from the XXX Institutional Data Access / Ethics Committee (contact via XXX) for researchers who meet the criteria for access to confidential data.*<br><br>*The data underlying the results presented in the study are available from (include the name of the third party*	All relevant data are within the manuscript and its Supporting Information files.

and contact information or URL).<br>• This text is appropriate if the data are owned by a third party and authors do not have permission to share the data.<br><br>* typeset	
Additional data availability information:	

Powered by Editorial Manager® and ProduXion Manager® from Aries Systems Corporation

Manuscript Click here to access/download;Manuscript;manuscript2.docx

Title page

Sex-based differences in long-term outcomes after stroke: a meta-analysis

Xiumei Guo, MD[1,2], Yu Xiong, MD[2], Xinyue Huang, MD[2], Zhigang Pan, MD[2], Xiaodong Kang, MD[2], Chunhui Chen, MD[2], Jianfeng Zhou, MD[2], Hanlin Zheng, MD[2], Yuping Chen, MD[2&], Weipeng Hu, MD[2&], Lingxing Wang, MD[1&], Feng Zheng, MD[2&]

[1] Department of Neurology, the Second Affiliated Hospital, Fujian Medical University, Quanzhou, Fujian Province, China 362000

[2] Department of Neurosurgery, the Second Affiliated Hospital, Fujian Medical University, Quanzhou, Fujian Province, China 362000

[&] **Corresponding authors**

Prof. Feng Zheng

No. 34 North Zhongshan Road, Quanzhou, Fujian Province, 362000, China

Phone: +86 0595-26655286

E-mail: dr.feng.zheng@gmail.com

Prof. Lingxing Wang

No. 34 North Zhongshan Road, Quanzhou, Fujian Province, 362000, China

Phone: +86 8097-22788097

E-mail: lxing502@fjmu.edu.cn

Prof. Weipeng Hu

No. 34 North Zhongshan Road, Quanzhou, Fujian Province, 362000, China

Phone: +86 0595-26655286

E-mail: 1923307274@qq.com

Prof. Yuping Chen

No. 34 North Zhongshan Road, Quanzhou, Fujian Province, 362000, China

Phone: +86 13959709342

E-mail: 503106356@qq.com

Acknowledgements:

None

Sources of funding:

None

Disclosures:

The authors have declared that no competing interest exists.

Sex-based differences in long-term outcomes after stroke: a meta-analysis

Abstract

Background: There is limited data on sex-related disparities in the long-term outcomes after stroke. We aim to investigate whether there are sex-based differences in long-term outcomes using pooled data.

Methods: Three databases (PubMed, Embase, and Cochrane Library) were systematically searched from inception to July 2022. This meta-analysis was performed in accordance with the recommendations and guidelines of the Preferred Reporting Items for Systematic Reviews and Meta-Analyses. The modified Newcastle-Ottawa scale was used to assess the risk of bias. In addition, a random-effects model was used.

Results: Twenty-two cohort studies with 84538 patients were included. There were 50.2% men and 49.8% women. Women had a higher mortality at 1 (odds ration [OR], 0.82; 95% confidence interval [CI][0.69, 0.99], P=0.03) and 10 (OR 0.72, 95% CI[0.65, 0.79], $P < 0.00001$) years, higher stroke recurrence at 1 year (OR 0.85, 95% CI[0.73, 0.98], P=0.02), lower favorable outcome at 1 year (OR 1.36, 95% CI[1.24, 1.49], $P < 0.00001$). No significant difference was detected between men and women in the outcomes of health-related quality of life and depression.

Conclusion: In this meta-analysis, the mortality at 1- and 5-year and stroke recurrence rates were higher in female patients than in male patients after stroke. In addition, females tended to experience less favorable outcomes in the first year after stroke. Finally, further long-term studies on sex disparities in stroke prevention, care, and management are warranted to explore the opportunities to reduce this gap.

Key words

Male, Female, Stroke, Long-term outcomes

1. Introduction

Stroke is a leading cause of mortality and disability worldwide, with 5.5 million deaths and 116.4 million disability-adjusted life-years [1], leading to substantial treatment costs for stroke care [2]. Stroke affects female and male patients differently, although the sources and consequences of these differences are unclear [3, 4]. It is known that there are sex-based differences in various factors of stroke, including risk factors, clinical acute stroke symptoms, and treatment management [5]. Moreover, previous studies have reported sex-based differences in stroke outcomes, where females are more likely to have greater stroke severity and poorer outcomes than males [6-9]. These findings are largely based on studies that have assessed stroke outcomes at discharge or within the first few months post-onset [6, 7, 9]. However, data on the long-term outcomes after stroke remain scarce [10, 11].

Although reductions in stroke mortality have occurred in recent years [12], females continue to bear a disproportionate burden of stroke compared to males, and this remains a significant global concern [13]. Understanding sex-based differences in stroke epidemiology and outcomes is important for reducing potential disparities and the excess burden of disability [10]. However, despite increasing evidence and interest in sex-based disparities in health, the available data on sex-related differences in long-term outcomes after stroke are limited [10, 11]. Therefore, this meta-analysis was performed to compare various long-term stroke outcomes between males and females to investigate whether there is

a sex-based disparity in long-term stroke outcomes and provide insights for improving stroke care and management.

2. Methods

2.1 Search strategy

This meta-analysis was performed in accordance with the recommendations and guidelines of the Preferred Reporting Items for Systematic Reviews and Meta-Analyses (PRISMA).[14] Two authors (X.G. and F.Z.) independently performed a comprehensive literature search. Studies were retrieved from the PubMed, Embase, and Cochrane databases from inception to August 2022, with no language restrictions. The complete search strategy is provided in the Supplementary Materials section. Additionally, the reference lists of the included studies were manually searched to identify additional relevant publications. All studies that met the inclusion criteria, as described below, were included in the analysis. Any discrepancies discovered during the literature search were resolved through consultation with the corresponding author (F.Z.).

2.2 Outcomes

Death, stroke recurrence, and favorable outcomes were the primary outcomes. Death was defined as all-cause mortality. Stroke recurrence was self-reported and defined as the aggravation of the original symptoms, the appearance of new signs, or re-hospitalization with a hospital admission diagnosis of recurrent stroke. Favorable outcomes referred to an individual's ability to perform activities of daily living. In stroke, this outcome is closely related to the severity and type of neurological impairments. The modified Rankin scale (mRS) was used to assess functional outcomes

and independent living.[15] The mRS is an ordinal scale ranging from 0–6, with 0 indicating no symptoms and 6 indicating patient death. A favorable outcome was defined as an mRS score of 0–2. Restoring favorable outcomes is a focus of stroke rehabilitation as they are strongly associated with independent living.

The secondary outcomes included health-related quality of life (HRQoL) and depression. The World Health Organization defines “quality of life” as an “individual’s perception of their position in life in the context of the culture and value systems in which they live in relation to their goals and expectations”[16]. HRQoL instruments commonly comprise the physical, social, and mental domains, aligning with the notion that health is a state of complete well-being and not just an absence of disease. The 36-Item Short Form Health Survey (SF-36) is the most commonly used instrument to measure HRQoL [17]. Depression was defined as a Hospital Anxiety subscale score of ≥11 [18].

2.3 Study selection

Two researchers (X. G. and Y. X.) independently screened the retrieved literature. The inclusion criteria for selecting the studies were as follows: (a) randomized controlled trials or observational cohort studies; (b) studies comparing long-term stroke outcomes among female and male patients with stroke; and (c) studies that reported at least one outcome. Exclusion criteria were as follows: (a) studies that used non-human subjects; (b) studies from which data could not be extracted; (c) non-comparative studies, including case reports, reviews, conference abstracts, letters, surveys, or satisfaction studies; (d) studies only equipped with a single-arm design; and (e) studies that did not meet the inclusion criteria.

2.4 Data extraction

Two authors (X.G. and Y.X.) independently extracted the baseline characteristics and primary and secondary outcomes from the included studies. Baseline data included the first author, year of publication, sample size, study design (hospital-based or population-based), age, the subtype of stroke, potential risk factors, stroke severity, and pre-stroke health. Any questions were communicated and resolved by the corresponding author (F.Z.).

2.5 Critical appraisal

Two authors (X. G. and Y. X.) independently assessed the quality of the included studies. The Cochrane risk bias assessment tool was used to assess the bias in randomized controlled studies [19]. The degree of bias risk (low, unknown, and high) was evaluated across seven aspects (random sequence generation, allocation concealment, blinding of researchers and participants, the integrity of outcome data, blind evaluation of research outcomes, selective reporting of research results, and other sources of bias) to reflect the quality of each study. The Newcastle Ottawa Scale, which assigns a score out of a possible of nine stars, was used to assess the quality of the observational cohort studies included in this meta-analysis [20]. The Scale rates were studied under the selection, comparability, and outcome categories. Four stars were available in the selection category, two in the comparability category, and three possible stars could be achieved in the outcome category. The maximum number of stars was nine, and studies were graded as “high quality,” with scores of ≥6, “moderate” with scores of

4–5, and “weak,” with scores of 0–3. A Newcastle Ottawa Scale score >7 indicated high quality. The quality of the included studies was evaluated carefully, and any differences in opinions were resolved through discussion.

2.6 Statistical analysis

This study assessed the differences between males and females in the following outcomes: mortality, favorable outcomes, stroke recurrence, HRQoL, and depression. Categorical variables were analyzed using the dominance odds ratio (OR), and continuous variables were analyzed using the mean difference (MD). In addition, a random effects model was used to pool the results of the included studies.

Statistical analysis was performed using the Review Manager software (version 5.4; The Cochrane Collaboration,2020; Nordic Cochrane Center, Copenhagen, Denmark). Dichotomous data were summarized using odds ratios (OR) and 95% confidence intervals (CI). Continuous data are displayed as MD and standard deviation (SD). Where appropriate, the SD was calculated based on the reported standard errors. Statistical significance was set at P-values < 0.05. Statistical heterogeneity was measured using c^2 and I^2 statistics. According to the recommendation of the Cochrane Statistical Methods Group [21], a significance level of heterogeneity was set at a P-value of 0.1, and the I^2 statistic was interpreted as follows: 0–40%, low heterogeneity; 30–60%, moderate heterogeneity; 50–90%, substantial heterogeneity; and 75–100%, considerable heterogeneity. Statistically significant heterogeneity was present at $P<.1$ and $I^2>50\%$. In these cases, sensitivity analysis and subgroup analysis were performed to assess the robustness of the results.

3. RESULTS

3.1 Study inclusion

A total of 1636 publications were retrieved, of which 1452 were obtained after removing duplicate studies. After screening titles and articles, a total of 1225 articles were excluded. The full text of the remaining 227 articles was assessed for eligibility, resulting in the exclusion of 154 studies with outcomes not stratified by sex and 37 conference abstracts without a complete text. Three studies were excluded because their data were not extractable. Fifteen of the remaining articles were excluded due to a lack of long-term outcome data based on sex-based differences. In total, 22 studies [10, 11, 22-41] comprising a total of 84538 patients (42431 and 42107 in the male and female groups, respectively) comparing long-term clinical outcomes after stroke were included in the present analysis. A flowchart of the search strategy is shown in Figure 1.

Until the literature search, no randomized controlled trials had been published on this topic. Hence, all 22 [10, 11, 22-41] articles were cohort studies (14 hospital-based studies and 8 population-based studies). The details of these studies are summarized in Table 1.

3.2 Primary outcomes

3.2.1 Mortality

First-year mortality was recorded in 13 studies [11, 28-33, 37-39, 41-43]. Pooled results showed a significant difference in first-year mortality between male and female stroke patients, in favor of male patients (odds ratio [OR], 0.82; 95% confidence interval [CI] 0.69% to 0.99%; *P*=.03) (Figure 2A).

Since the data based on ischemic stroke were sufficiently provided in eight studies [11, 28, 31-33, 38, 41, 43], further subgroup analysis was performed on the outcome of the 1-year mortality, with no significant difference detected in the ischemic stroke subgroup (OR 0.83, 95% CI 0.67% to 1.03%, *P*=.09) (Supplement Figure 1A). Four studies [10, 22, 23, 29] recorded data assessing mortality between males and females over 5 years. There was no significant difference between the two groups (OR 0.87, 95% CI 0.57% to 1.32%, *P*=.52) (Figure 2B). Due to substantial heterogeneity (*P*=.003, I^2=78%), a sensitivity analysis was employed to assess the robustness of the findings. After excluding one study [22], significant differences were detected in the pooled results (OR 0.68, 95% CI 0.63% to 0.75%, *P*<.00001), without substantial heterogeneity (*P*=.98, I^2=0%). The 10-year mortality was recorded in two studies [10, 36], with a significant difference in favor of males (OR 0.72, 95% CI 0.65% to 0.79%, *P*<.00001) (Figure 2C).

3.2.2 Favorable outcome

Four of the included studies [11, 29-31] investigated favorable outcomes 1 year after stroke. Compared with males, females had significantly lower favorable outcomes (OR 1.36, 95% CI 1.24% to 1.49%, *P*<.00001) (Figure 2D). Further subgroup analysis of patients with ischemic stroke showed a greater tendency for 1-year favorable outcomes in male patients than in female patients (OR 1.36, 95% CI 1.23% to 1.50%, *P*<.0001) (Supplement Figure 1B). Regarding 5-year favorable outcomes, no significant difference was detected between males and females (OR 2.88, 95% CI 0.21% to 38.63%, *P*=.43) (Figure 2E).

3.2.3 Recurrent stroke

Six studies [11, 29, 32, 34, 41, 44] reported the outcome of 1-year recurrent stroke, with significant differences detected in 1-year stroke recurrence between males and females (OR 0.85, 95% CI 0.73% to 0.98%, *P*=.02) (Figure 2F) in favor of males. Subgroup analysis of ischemic stroke showed no significant difference in 1-year recurrent stroke between males and females (OR 0.98, 95% CI 0.75% to 1.28%, *P*=.87) (Supplementary Figure 1C). The recurrence rate did not differ between males and females in the 5-year recurrence rates (OR 1.09, 95% CI 0.83% to 1.43%; *P*=.53) (Figure 2G) and in the 10-year stroke recurrence rates (OR 0.96, 95% CI 0.76% to 1.21%, *P*=.72) (Figure 2H).

3.3 Second outcomes

3.3.2 HRQoL

Two studies (26, 35) reported HRQoL outcomes, with no significant difference detected between male and female patients in physical health (OR -2.18, 95% CI -12.18% to 7.81%, *P*=.67) (Figure 3A) and mental health (OR 0.39, 95% CI -2.35% to 3.13%, *P*=.78) (Figure 3B).

3.3.3 Depression

The incidence of depression was reported in three studies [10, 24, 44]. No significant difference was detected between male and female patients with stroke (OR 0.39, 95% CI -2.35% to 3.13%, *P*=.78) (Figure 3C).

4. DISCUSSION

Our meta-analysis revealed several clinically relevant findings. First, regarding long-term

outcomes after stroke, female patients had significantly higher 1-year and 10-year mortality rates and higher recurrence rates in the first year after stroke. In addition, females were less likely to achieve favorable outcomes in their first year. There was no significant difference in self-reported outcomes, including HRQoL and depression, as indicated by the analysis (Figure 4).

There was no significant difference in 5-year mortality between male and female patients, but there was substantial heterogeneity. Sensitivity analysis was employed according to the Cochrane Handbook for Systematic Reviews of Interventions [19] to obtain robust results. After removing the study by Adoukonou et al. [22], the heterogeneity significantly decreased, with higher 5-year mortality detected in female patients. This may be due to the high proportion of patients (over 17%) who were lost to follow-up in the study by Adoukonou et al., which may have biased the findings.

Several factors are associated with mortality. In this meta-analysis, female patients with stroke tended to be older, had higher pre-stroke handicaps, and were more serious, with higher National Institutes of Health Stroke Scale (NIHSS) scores. The studies by Xu et al. and Wang et al.[10, 11] showed that although female patients were associated with statistically higher mortality, after adjusting for relevant confounders, including age, stroke severity, stroke subtype, and other comorbidities, mortality in male patients may be higher. Among these factors, Wang et al.[11] believe that age may be one of the most important causes since a significant difference showed that females had lower mortality rates than males of older age. However, owing to the lack of sufficient data based on different ages, further subgroup analysis could not be performed in the present study. In addition, the subgroup analysis of ischemic stroke showed comparable mortality between males and females in the first year after stroke.

Compared with female patients, more favorable outcomes were observed in male patients 1 year

after stroke, which became insignificant 5 years after stroke. A subgroup analysis was performed considering the significant influence of the stroke subtype. Pooled data regarding ischemic stroke showed that male patients had more favorable outcomes at 1 year, which was consistent with the primary outcome in the present study. Preclinical and clinical evidence suggests that a combination of factors, including exposure to sex hormones, sex chromosomes, and brain and vascular microenvironments, may contribute to sex-based differences in the cellular mechanisms underlying stroke injury [45]. Furthermore, a study by Wang et al.[11] showed that older females, especially those >65 years of age, were more prone to have poor outcomes. This may be explained by age-dependent differences between females and males, such as atrial fibrillation. The risk of atrial fibrillation-associated stroke increased with age and was higher in females than in males [46].

The present study detected a significant difference in 1-year stroke recurrence in favor of male patients. However, this difference became insignificant after 5 and 10 years. Moreover, it was reported that there was no significant difference in stroke recurrence when adjusting for confounders such as age, pre-stroke dependency, and stroke severity [10, 41]. Interestingly, in a recent study using a crude model, a significant difference in the outcome of one-year stroke recurrence was detected in male patients [11], which was consistent with our findings. However, this difference was attenuated when accounting for blood pressure and serum-related covariates.

In our meta-analysis, female patients had poorer long-term HRQoL than male patients in terms of both physical and mental health. However, it is difficult to pool the data with consistent standards because different measurements were utilized to define the outcomes of quality of life, depression, anxiety, activities of daily living, and cognitive impairment. Therefore, future studies using a unified measurement and formula mode are warranted to further compare these outcomes between male and

female patients.

Previous studies [27, 47, 48] reported inconsistent results regarding sex differences in the prognosis of patients with stroke. However, in the present meta-analysis, females tended to have poorer outcomes, higher stroke recurrence in the first year after stroke, and higher mortality in the first and tenth years. Therefore, it is necessary to focus on potential sex-based differences in managing long-term health care in patients with stroke.

This meta-analysis had some limitations. First, all studies included in the present analysis were cohort studies. Among the included studies, 15 were conducted retrospectively, and any conclusions drawn were consequently subject to the limitations of the retrospective study design, including recall and observer bias. In addition, 14 studies were hospital-based, which may have introduced selection bias. More population-based studies are warranted to further examine sex-based differences in the long-term outcomes after stroke. Second, subgroup analysis on stroke subtypes other than ischemic stroke was not possible in the present study due to a lack of patient-level data. Future studies are warranted to explore sex disparities in patients with different types of stroke.

5. CONCLUSION

This meta-analysis found that mortality at 1-year and 5-years, as well as stroke recurrence rates, were higher in female patients than in male patients after stroke. In addition, females tend to experience less favorable outcomes in the first year after a stroke. Further long-term studies on sex disparities in stroke prevention, care, and management are warranted to explore the opportunities to reduce this gap.

6. REFERENCES

1. Global, regional, and national burden of neurological disorders, 1990-2016: a systematic analysis for the Global Burden of Disease Study 2016. Lancet Neurol. 2019;18(5):459-80.

2. Patel A, Berdunov V, Quayyum Z, King D, Knapp M, Wittenberg R. Estimated societal costs of stroke in the UK based on a discrete event simulation. Age Ageing. 2020;49(2):270-6.

3. Bonkhoff AK, Karch A, Weber R, Wellmann J, Berger K. Female Stroke: Sex Differences in Acute Treatment and Early Outcomes of Acute Ischemic Stroke. Stroke. 2021;52(2):406-15.

4. Dehlendorff C, Andersen KK, Olsen TS. Sex Disparities in Stroke: Women Have More Severe Strokes but Better Survival Than Men. J Am Heart Assoc. 2015;4(7).

5. Ali M, van Os HJA, van der Weerd N, Schoones JW, Heymans MW, Kruyt ND, et al. Sex Differences in Presentation of Stroke: A Systematic Review and Meta-Analysis. Stroke. 2022;53(2):345-54.

6. Gall S, Phan H, Madsen TE, Reeves M, Rist P, Jimenez M, et al. Focused Update of Sex Differences in Patient Reported Outcome Measures After Stroke. Stroke. 2018;49(3):531-5.

7. Lisabeth LD, Reeves MJ, Baek J, Skolarus LE, Brown DL, Zahuranec DB, et al. Factors influencing sex differences in poststroke functional outcome. Stroke. 2015;46(3):860-3.

8. Phan HT, Blizzard CL, Reeves MJ, Thrift AG, Cadilhac DA, Sturm J, et al. Sex Differences in Long-Term Quality of Life Among Survivors After Stroke in the INSTRUCT. Stroke. 2019;50(9):2299-306.

9. Palnum KD, Andersen G, Ingeman A, Krog BR, Bartels P, Johnsen SP. Sex-related differences in quality of care and short-term mortality among patients with acute stroke in

Denmark: a nationwide follow-up study. Stroke. 2009;40(4):1134-9.

10. Xu M, Amarilla Vallejo A, Cantalapiedra Calvete C, Rudd A, Wolfe C, O'Connell MDL, et al. Stroke Outcomes in Women: A Population-Based Cohort Study. Stroke. 2022:101161STROKEAHA121037829.

11. Wang M, Wang CJ, Gu HQ, Meng X, Jiang Y, Yang X, et al. Sex Differences in Short-Term and Long-Term Outcomes Among Patients With Acute Ischemic Stroke in China. Stroke. 2022;53(7):2268-75.

12. Goldstein LB. Introduction for Focused Updates in Cerebrovascular Disease. Stroke. 2020;51(3):708-10.

13. Lundberg GP, Volgman AS. Burden of stroke in women. Trends Cardiovasc Med. 2016;26(1):81-8.

14. Page MJ, McKenzie JE, Bossuyt PM, Boutron I, Hoffmann TC, Mulrow CD, et al. The PRISMA 2020 statement: an updated guideline for reporting systematic reviews. Bmj. 2021;372:n71.

15. Ernst M, Boers AMM, Forkert ND, Berkhemer OA, Roos YB, Dippel DWJ, et al. Impact of Ischemic Lesion Location on the mRS Score in Patients with Ischemic Stroke: A Voxel-Based Approach. AJNR Am J Neuroradiol. 2018;39(11):1989-94.

16. Development of the World Health Organization WHOQOL-BREF quality of life assessment. The WHOQOL Group. Psychol Med. 1998;28(3):551-8.

17. Anderson C, Laubscher S, Burns R. Validation of the Short Form 36 (SF-36) health survey questionnaire among stroke patients. Stroke. 1996;27(10):1812-6.

18. Zigmond AS, Snaith RP. The hospital anxiety and depression scale. Acta Psychiatr

Scand. 1983;67(6):361-70.

19. Cumpston M, Li T, Page MJ, Chandler J, Welch VA, Higgins JP, et al. Updated guidance for trusted systematic reviews: a new edition of the Cochrane Handbook for Systematic Reviews of Interventions. The Cochrane database of systematic reviews. 2019;10:ED000142.

20. Deeks JJ, Dinnes J, D'Amico R, Sowden AJ, Sakarovitch C, Song F, et al. Evaluating non-randomised intervention studies. Health Technol Assess. 2003;7(27):iii-x, 1-173.

21. Colby GP, Paul AR, Radvany MG, Gandhi D, Gailloud P, Huang J, et al. A single center comparison of coiling versus stent assisted coiling in 90 consecutive paraophthalmic region aneurysms. J Neurointerv Surg. 2012;4(2):116-20.

22. Adoukonou T, Agbétou M, Bangbotché R, Kossi O, Fotso Mefo P, Magne J, et al. Long-Term Mortality of Stroke Survivors in Parakou: 5-Year Follow-Up. Journal of Stroke and Cerebrovascular Diseases. 2020;29(6).

23. Akhtar N, Kate M, Kamran S, Singh R, Bhutta Z, Saqqur M, et al. Sex-Specific Differences in Short-Term and Long-Term Outcomes in Acute Stroke Patients from Qatar. Eur Neurol. 2020;83(2):154-61.

24. Carod-Artal J, Egido JA, González JL, Varela de Seijas E. Quality of life among stroke survivors evaluated 1 year after stroke: experience of a stroke unit. Stroke. 2000;31(12):2995-3000.

25. de Ridder I, Kuramatsu J, Gerner S, Madžar D, Lücking H, Kloska S, et al. No sex differences in long-term functional outcome after intracerebral hemorrhage. Int J Stroke. 2017;12(4):416-20.

26. Feigin VL, Barker-Collo S, Parag V, Senior H, Lawes CM, Ratnasabapathy Y, et al.

Auckland Stroke Outcomes Study. Part 1: Gender, stroke types, ethnicity, and functional outcomes 5 years poststroke. Neurology. 2010;75(18):1597-607.

27. Gall SL, Tran PL, Martin K, Blizzard L, Srikanth V. Sex differences in long-term outcomes after stroke: functional outcomes, handicap, and quality of life. Stroke. 2012;43(7):1982-7.

28. Geng C, Lin Y, Tang Q, Tang Y, Wang X, Zhou JS, et al. Sex differences in clinical characteristics and 1-year outcomes of young ischemic stroke patients in east China. Therapeutics and Clinical Risk Management. 2019;15:33-8.

29. Jönsson AC, Ek J, Kremer C. Outcome of men and women after atrial fibrillation and stroke. Acta Neurol Scand. 2015;132(2):125-31.

30. Kim JS, Lee KB, Roh H, Ahn MY, Hwang HW. Gender differences in the functional recovery after acute stroke. J Clin Neurol. 2010;6(4):183-8.

31. Kong FY, Tao WD, Hao ZL, Liu M. Predictors of one-year disability and death in Chinese hospitalized women after ischemic stroke. Cerebrovasc Dis. 2010;29(3):255-62.

32. Li B, Wang T, Lou Y, Guo X, Gu H, Zhu Y, et al. Sex Differences in Outcomes and Associated Risk Factors After Acute Ischemic Stroke in Elderly Patients: A Prospective Follow-up Study. J Stroke Cerebrovasc Dis. 2015;24(10):2277-84.

33. Medlin F, Amiguet M, Eskandari A, Michel P. Sex differences in acute ischaemic stroke patients: clinical presentation, causes and outcomes. Eur J Neurol. 2020;27(8):1680-8.

34. Mohan KM, Crichton SL, Grieve AP, Rudd AG, Wolfe CD, Heuschmann PU. Frequency and predictors for the risk of stroke recurrence up to 10 years after stroke: the South London Stroke Register. J Neurol Neurosurg Psychiatry. 2009;80(9):1012-8.

35. Patel MD, McKevitt C, Lawrence E, Rudd AG, Wolfe CD. Clinical determinants of long-

term quality of life after stroke. Age Ageing. 2007;36(3):316-22.

36. Prencipe M, Culasso F, Rasura M, Anzini A, Beccia M, Cao M, et al. Long-term prognosis after a minor stroke: 10-Year mortality and major stroke recurrence rates in a hospital-based cohort. Stroke. 1998;29(1):126-32.

37. Sarfo FS, Akassi J, Kyem G, Adamu S, Awuah D, Kantanka OS, et al. Long-Term Outcomes of Stroke in a Ghanaian Outpatient Clinic. J Stroke Cerebrovasc Dis. 2018;27(4):1090-9.

38. Sheikh K, Bullock CM. Effect of measurement on sex difference in stroke mortality. Stroke. 2007;38(3):1085-7.

39. Shen Q, Cordato D, Chan DK, Hung WT, Karr M. Identifying the determinants of 1-year post-stroke outcomes in elderly patients. Acta Neurol Scand. 2006;113(2):114-20.

40. Vemmos KN, Bots ML, Tsibouris PK, Zis VP, Takis CE, Grobbee DE, et al. Prognosis of stroke in the south of Greece: 1 year mortality, functional outcome and its determinants: the Arcadia Stroke Registry. J Neurol Neurosurg Psychiatry. 2000;69(5):595-600.

41. Wang Z, Li J, Wang C, Yao X, Zhao X, Wang Y, et al. Gender differences in 1-year clinical characteristics and outcomes after stroke: results from the China National Stroke Registry. PLoS One. 2013;8(2):e56459.

42. de Ridder IR, Fransen PS, Beumer D, Berkhemer OA, van den Berg LA, Wermer MJ, et al. Is Intra-Arterial Treatment for Acute Ischemic Stroke Less Effective in Women than in Men? Interv Neurol. 2016;5(3-4):174-8.

43. Vemmos KN, Takis CE, Georgilis K, Zakopoulos NA, Lekakis JP, Papamichael CM, et al. The Athens stroke registry: results of a five-year hospital-based study. Cerebrovasc Dis.

2000;10(2):133-41.

44. Gall SL, Tran PL, Martin K, Blizzard L, Srikanth V. Sex differences in long-term outcomes after stroke: Functional outcomes, handicap, and quality of life. Stroke. 2012;43(7):1982-7.

45. Chauhan A, Moser H, McCullough LD. Sex differences in ischaemic stroke: potential cellular mechanisms. Clin Sci (Lond). 2017;131(7):533-52.

46. Lang C, Seyfang L, Ferrari J, Gattringer T, Greisenegger S, Willeit K, et al. Do Women With Atrial Fibrillation Experience More Severe Strokes? Results From the Austrian Stroke Unit Registry. Stroke. 2017;48(3):778-80.

47. Phan HT, Reeves MJ, Blizzard L, Thrift A, Cadilhac D, Heeley E, et al. Sex differences n long-term mortality and disability after stroke: The international stroke outcomes study. Stroke. 2016;47.

48. Silva GS, Lima FO, Camargo EC, Smith WS, Lev MH, Harris GJ, et al. Gender differences in outcomes after ischemic stroke: role of ischemic lesion volume and intracranial large-artery occlusion. Cerebrovasc Dis. 2010;30(5):470-5.

Table 1 Baseline characteristic for included studies

Author and publication year	Study design and period	Total (n)	Patients(n)		Age		Subtype(%)		Stroke severity†		Smoke (%)		Hypertension (%)		Diabetes (%)		AF (%)		IHD (%)		Dyslipidemia (%)		Drink (%)		Pre-stroke status§		NOS
			M	F	M	F	M	F	M	F	M	F	M	F	M .	F	M	F	M	F	M	F	M	F	M	F	
Akhtar<br>2020 (23)	H-B<br>2014-2019	819	519	372	62.9±14.1	65.9±14.2	IS 71.7<br>ICH 13.7<br>TIA 14.6	IS 70.2<br>ICH 13.7<br>TIA 16.1	2(0-4)	1(0-3)	32.4	0.8	83.4	82.5	68.2	74.7	8.9	14.0	20.2	11.8	51.8	55.1	NA	NA	NA	NA	7
Feigin<br>2010 (26)	P-B<br>2007-2008	418	217	201	64.1±4.1	75.5±2.5	IS 80.4*<br>ICH 8.1<br>UND 5.0		4.1(4.3)	4.7(4.9)	NA	NA	NA	NA	NA	NA	NA	NA	NA	NA	NA	NA	NA	NA	NA	NA	8
Geng<br>2019 (28)	H-B<br>2004-2008	228	147	81	42.78±6.0	42.3±6.6	IS 100	IS 100	4(2,10)	4(1,8)	51.7	5.0	34.0	19.8	8.8	5.0	5.4	14.8	0.7	1.0	5.4	1.0	37.4	2.5	NA	NA	7
Kim<br>2010 (30)	H-B<br>2005-2009	1055	575	480	64.83±11.98	70.09±13.02	IS 87.2<br>TIA 5.7<br>ICH 7.1	IS 86.9<br>TIA 4 .8<br>ICH8.3	Mild 87.1<br>Moderate10.2<br>Severe7.3.	Mild 82<br>Moderate7.7<br>Severe5.2	46.9	6.8	60.1	62.6	33.3	29.2	NA	NA	NA	NA	16.1	22.5	NA	NA	NA	NA	8
Prencipe<br>1998 (36)	H-B<br>1077-1986	322	244	78	55*		NA	NA	NA	NA	NA	NA	54*		17*		NA	NA	NA	NA	42	NA	NA	NA	NA	NA	8
Ridder<br>2016 (25)	H-B<br>2006-2014	823	443	380	70(12)	73(12)	ICH 100	ICH 100	12(5-26)	15(6-28)	NA	NA	83	78	NA	NA	24	19	NA	NA	NA	NA	NA	NA	0(0-2)	1(0-2)	8
Sarfo<br>2017 (37)	H-B<br>2012-2014	607	305	302	59.9±13,9*		IS 40.3*<br>ICH 18.6<br>UND 41.1		NA	NA	NA	NA	NA	NA	NA	NA	NA	NA	NA	NA	NA	NA	NA	NA	NA	NA	7
Xu<br>2022 (10)	P-B<br>1995-2019	6687	3438	3249	0-64y:42<br>65-74y:26<br>75-84y:27<br>>85y:9	0-64y:26<br>65-74y:21<br>74-84y:23<br>>85y:710	IS 70<br>ICH 13<br>SH 4<br>UNC 2<br>UND 10	IS 71<br>ICH 11<br>SH 5<br>UNC 4<br>UND 9	Mild 28<br>Moderate 29<br>Severe 6<br>Unknown 37	Mild 22<br>Moderate 31<br>Severe 8<br>Unknown 40	66	42	64	67	22	21	15	20	20	19	30	28	NA	NA	5	12	9
Wang<br>2022 (11)	P-B<br>2015-2018	9038	6236	2802	61.26±11.42	64.78±10.84	IS 100	IS 100	4.43±4.2	4.85±4.44	45.1	3.43	68.13	60.89	22.77	27.09	6.59	9.74	9.77	13.13	8.07	8.57	NA	NA	0.42±0.88	0.44±0.92	9
Adoukonou<br>2020 (22)	H-B<br>2012-2018	247	136	111	58.1±13.4*		IS 36.8*<br>ICH 27.9<br>UND 35.2		NA	NA	NA	NA	NA	NA	NA	NA	NA	NA	NA	NA	NA	NA	NA	NA	NA	NA	6
Johnson<br>2015 (29)	H-B<br>2008-2009	155	94	61	79.5±8.7	84.8±8.1	ICH 85.2*		Mild 59<br>Moderate16<br>Major 25	Mild 47<br>Moderate17<br>Major 36	63	25	61	65	31	15	NA	NA	NA	NA	NA	NA	NA	NA	NA	NA	7
Vemmons<br>2000 (40)	P-B<br>1991-1992	152	44	108	NA	NA	IS 64.9<br>ICH 20.9<br>PAD 14.2	IS 75,7<br>ICH 12.5<br>PAD11.8	2(0-6)	2(0-5)	34	2.1	78.4	84.6	27.9	32.2	30.8	38.3	10.4	7.0	41.1	46.4	20.8	1.4	NA	NA	7
Kong<br>2010 (31)	H-B<br>2002-2007	2774	1655	1119	63±13.6	65±13.5	IS 100	IS 100	7.32	8.72	44.4	5.0	56.3	56.6	17.7	21.7	10.9	29.7	1.6	2.3	11.5	9.6	30.6	3.0	NA	NA	8
Cordato<br>2005 (24)	H-B<br>2002-2003	186	90	96	65-74.9y:15.4*<br>75-84.9y:28.4<br>≥85y:48.9		NA	NA	NA	NA	NA	NA	30.5*		30.3*		42.4*		29.2*		NA	NA	14.4	6	25.2	NA	7
Mohan<br>2009 (34)	P-B<br>1995-2004	2874	1427	1447	< 65y:30.0*<br>65-74y:26.6<br>75-84y:43.4		IS 72.7*<br>ICH 13.7<br>SH 6.0<br>UND 7.6		GCS < 8: 18.1*<br>GCS 9-12: 15.3<br>GCS 13-15: 66.6		35.6*		64.1*		18.0*		17.2*		11.7*		NA	NA	NA	NA	NA	NA	7
Medlin<br>2020 (33)	P-B<br>2003-2016	3993	2236	1757	69.9(20.4)	77(18.1)	IS 100	IS 100	6(10)	7(13)	28	18	71	72.4	21.3	15.3	25.5	33.7	NA	NA	NA	NA	NA	NA	0(1)	1(2)	8
Carod-Artal<br>2000 (24)	H-B<br>1996-1997	118	65	53	65.16(11.4)	71.80(8.88)	IS 89*<br>ICH 11		NA	NA	28.89*		65.56*		30*		26.67*				40	NA	NA	NA	NA	NA	6
Gall<br>2010 (27)	P-B<br>1995-1999	494	249	245	70.6(12.6)	73.7(14.4)	IS 85.9<br>ICH 12.1<br>SH 0.0<br>UND 2.0	IS 84.9<br>ICH 8.2<br>SH 0.4<br>UND 6.5	4.8(5.1)	5.6(5.7)	67	35	51.0	59.6	18.9	15.9	15.7	17.1	16.9	9.0	NA	NA	79.5	51.6	19.6(1.5)	19.1(2.7)	8

Table 1 continued.

Patel<br>2007 (35)	P-B<br>1995-1007	397	212	185	＜65y:34*<br>65-75y:33.5<br>>75y:32.5		NA	NA	NA	NA	NA	NA	71.8*		62*		18.4*		11.6*		NA	NA	NA	NA	BI<15 1.5*<br>BI 15-20 98.5		8
Li<br>2015 (32)	H-B<br>2009-2011	881	488	383	NA	NA	IS 100	IS 100	Mild 58.7<br>Moderate 27<br>Severe 14.3	Mild 56.1<br>Moderate 29.8<br>Severe 14.1	28.3	12.8	69.1	76.5	26.2	33.2	15.4	18.3	NA	NA	18.9	27.2	11.3	0.3	NA	NA	7
Sheikh<br>2007 (38)	P-B<br>1994-1997	40450	16391	24059	73.5	75.5	IS 57.1<br>ICH 9.16<br>UD 32.4	IS 55.1<br>ICH 9.12<br>UD 33.7	NA	NA	NA	NA	NA	NA	NA	NA	NA	NA	NA	NA	NA	NA	NA	NA	NA	NA	9
Wang<br>2013 (41)	H-B<br>2007-2008	11560	7118	4442	64±12.3	67.9±11.9	NA	NA	Mild 52.3<br>Moderate 27<br>Severe 10.5	Mild 45.3<br>Moderate 29.8<br>Severe 5.5	60.7	6.0	61.5	68.1	19.7	24.8	5.3	11.1	NA	NA	11.5	11.0	14.9	0.5	34.5	34.0	7

M, male; F, female; H-B, hospital-based; P-B, population-based; AF, atrial fibrillation; IHD, ischemic heart disease; IS, ischemic stroke; ICH, intracerebral hemorrhage; SH, subarachnoid hemorrhage; UND, undetermined; UNC, unclassified; GCS, Glasgow Coma Scale; NIHSS, National Institutes of Health Stroke Scale; BI, Barthel Index.

NOS: Newcastle Ottawa scale (NOS), which gives a score out of a possible total of 9 stars. Scale rates were studied under the categories of selection, comparability, and outcome. Four stars are available in the selection category, two stars are available in the comparability section, and three possible 3 stars can be achieved in the outcome categories.

* No separate value.

† Stroke severity was measured using GCS (Mohan) or NIHSS (remaining studies).

§ Pre-stroke status was measured using BI (Patel, Gall) or mRS (remaining studies)

Figure legends

Fig. 1 Flow chart showing the search strategy

Fig. 2 Forest plots showing primary outcomes between males and females. M-H Mantel-Haenszel statistic

Fig. 3 Forest plot showing secondary outcomes between males and females. M-H: Mantel-Haenszel statistic

Fig. 4 The comparison of long-term outcomes between males and females

Fig. 1 Flow chart showing the search strategy

Click here to access/download;Figure;3. figure 1.tiff

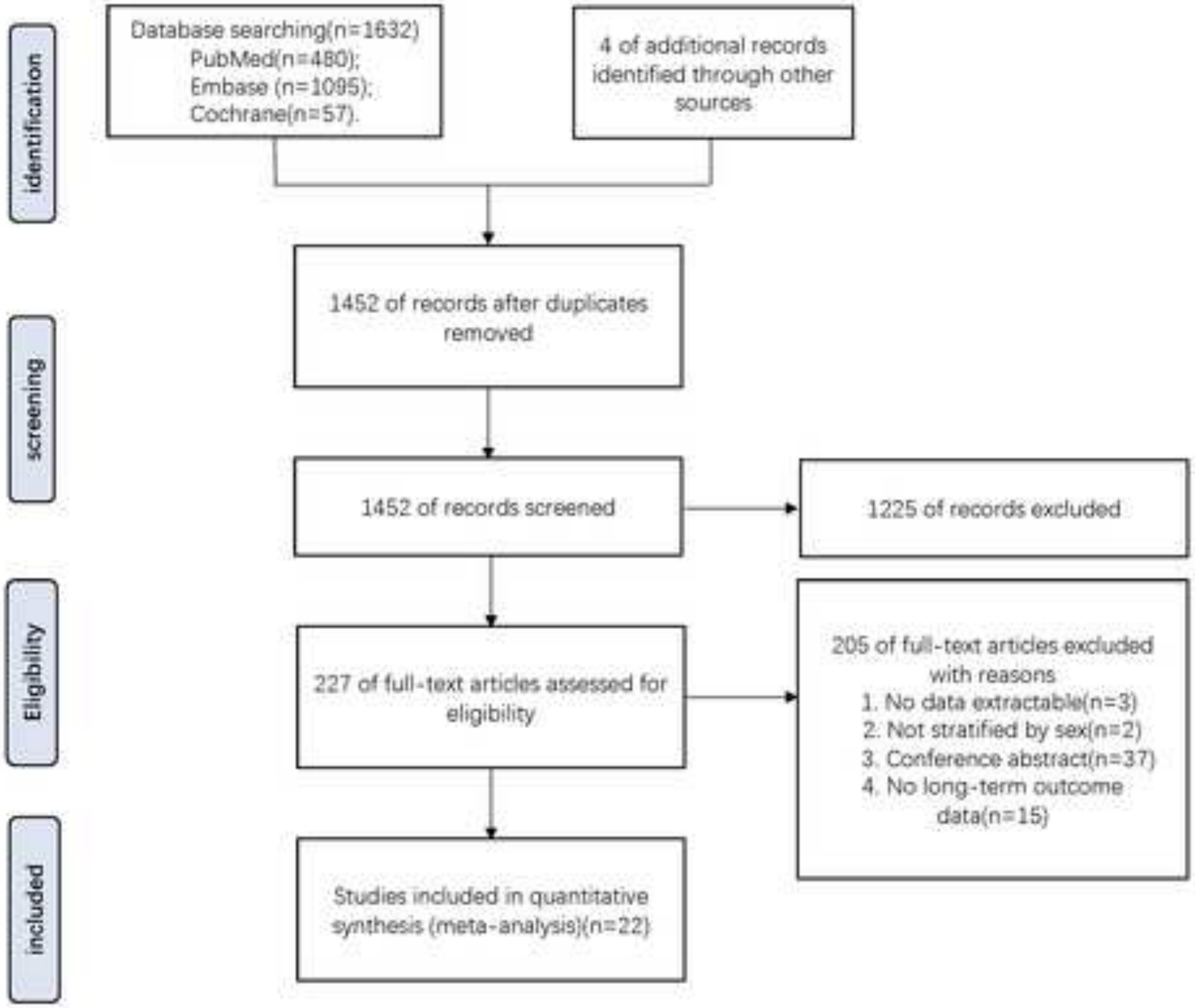

Fig. 2 Forest plots showing primary outcomes between males and females. M-H Mantel-Haenszel statistic

Click here to access/download;Figure;figure 2.tiff

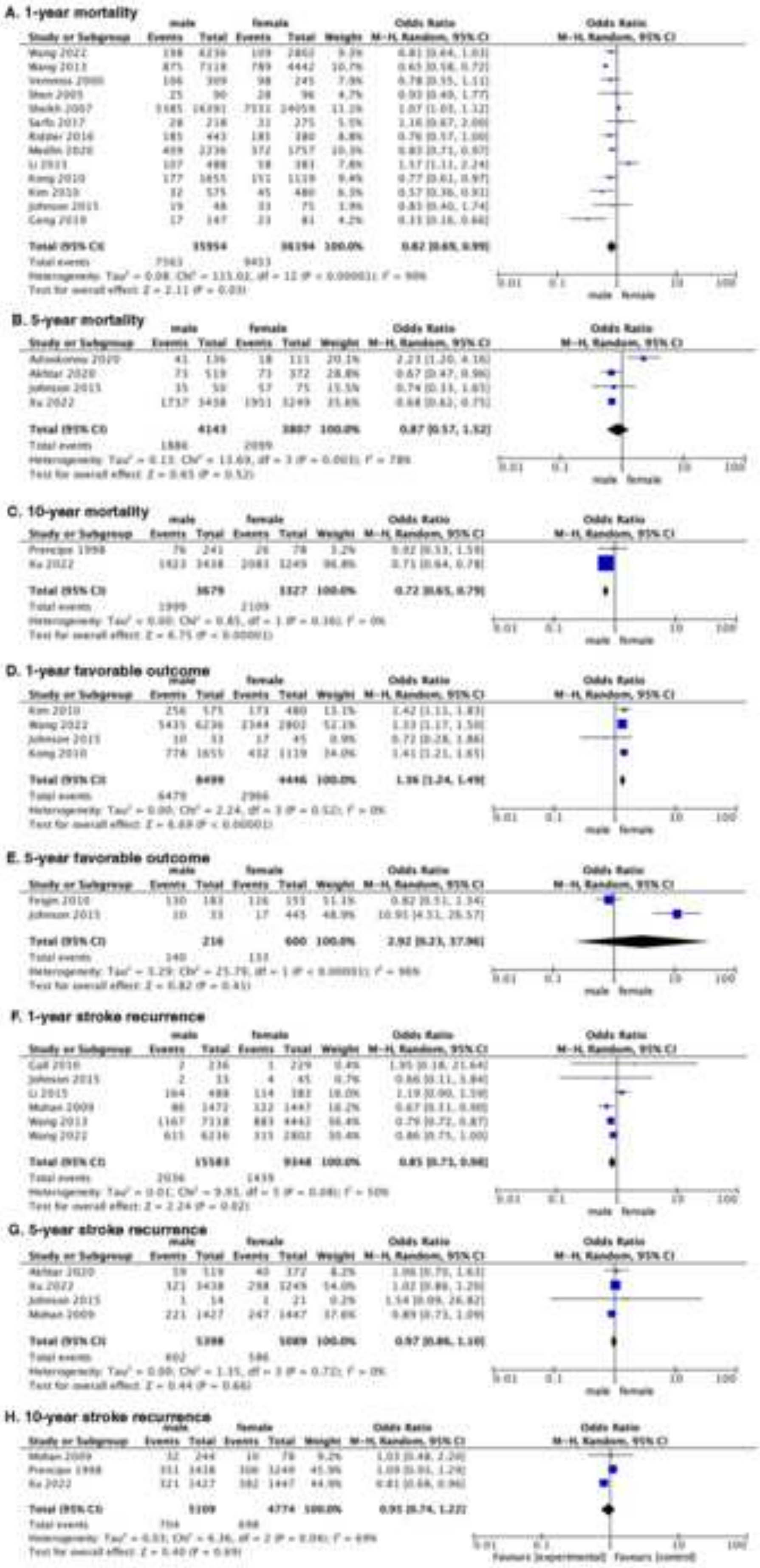

Fig. 3 Forest plot showing secondary outcomes between males and females. M-H: Mantel-Haenszel statistic

Click here to access/download;Figure;figure 3.tiff

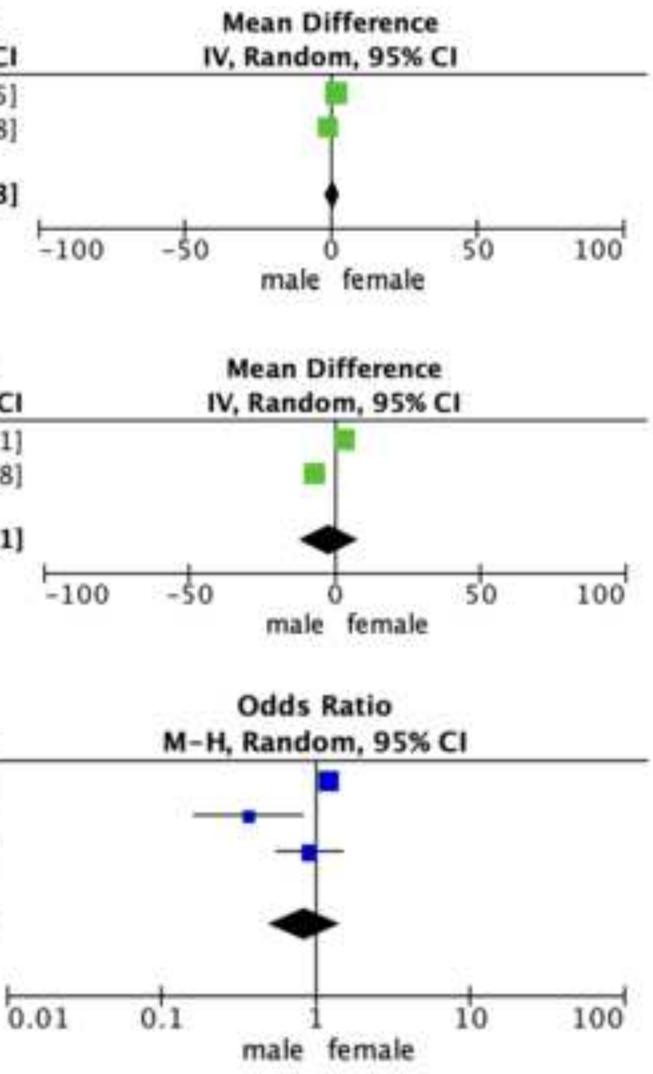

A. HRQoL (mental)

Study or Subgroup	male Mean	male SD	male Total	female Mean	female SD	female Total	Weight	Mean Difference IV, Random, 95% CI
Feigin 2010	52.1	9.6	217	50.4	10.7	201	53.1%	1.70 [-0.25, 3.65]
Patel 2007	46.1	12.4	212	47.2	11.8	185	46.9%	-1.10 [-3.48, 1.28]
**Total (95% CI)**			**429**			**386**	**100.0%**	**0.39 [-2.35, 3.13]**

Heterogeneity: $Tau^2 = 2.68$; $Chi^2 = 3.17$, df = 1 ($P = 0.07$); $I^2 = 68\%$
Test for overall effect: $Z = 0.28$ ($P = 0.78$)

B. HRQoL (physical)

Study or Subgroup	male Mean	male SD	male Total	female Mean	female SD	female Total	Weight	Mean Difference IV, Random, 95% CI
Feigin 2010	43.5	10.4	217	40.6	11.5	201	50.2%	2.90 [0.79, 5.01]
Patel 2007	28.3	12.4	212	35.6	12.2	185	49.8%	-7.30 [-9.72, -4.88]
**Total (95% CI)**			**429**			**386**	**100.0%**	**-2.18 [-12.18, 7.81]**

Heterogeneity: $Tau^2 = 50.68$; $Chi^2 = 38.73$, df = 1 ($P < 0.00001$); $I^2 = 97\%$
Test for overall effect: $Z = 0.43$ ($P = 0.67$)

C. Deppression

Study or Subgroup	male Events	male Total	female Events	female Total	Weight	Odds Ratio M-H, Random, 95% CI
Xu 2022	788	3438	642	3249	44.1%	1.21 [1.07, 1.36]
Carod-Artal 2000	36	64	42	54	22.7%	0.37 [0.16, 0.83]
Gall 2010	38	236	40	229	33.2%	0.91 [0.56, 1.48]
**Total (95% CI)**		**3738**		**3532**	**100.0%**	**0.84 [0.49, 1.45]**
Total events	862		724			

Heterogeneity: $Tau^2 = 0.17$; $Chi^2 = 9.18$, df = 2 ($P = 0.01$); $I^2 = 78\%$
Test for overall effect: $Z = 0.63$ ($P = 0.53$)

Fig. 4 The comparison of long-term outcomes between males and females

Click here to access/download;Figure;figure 4.tiff

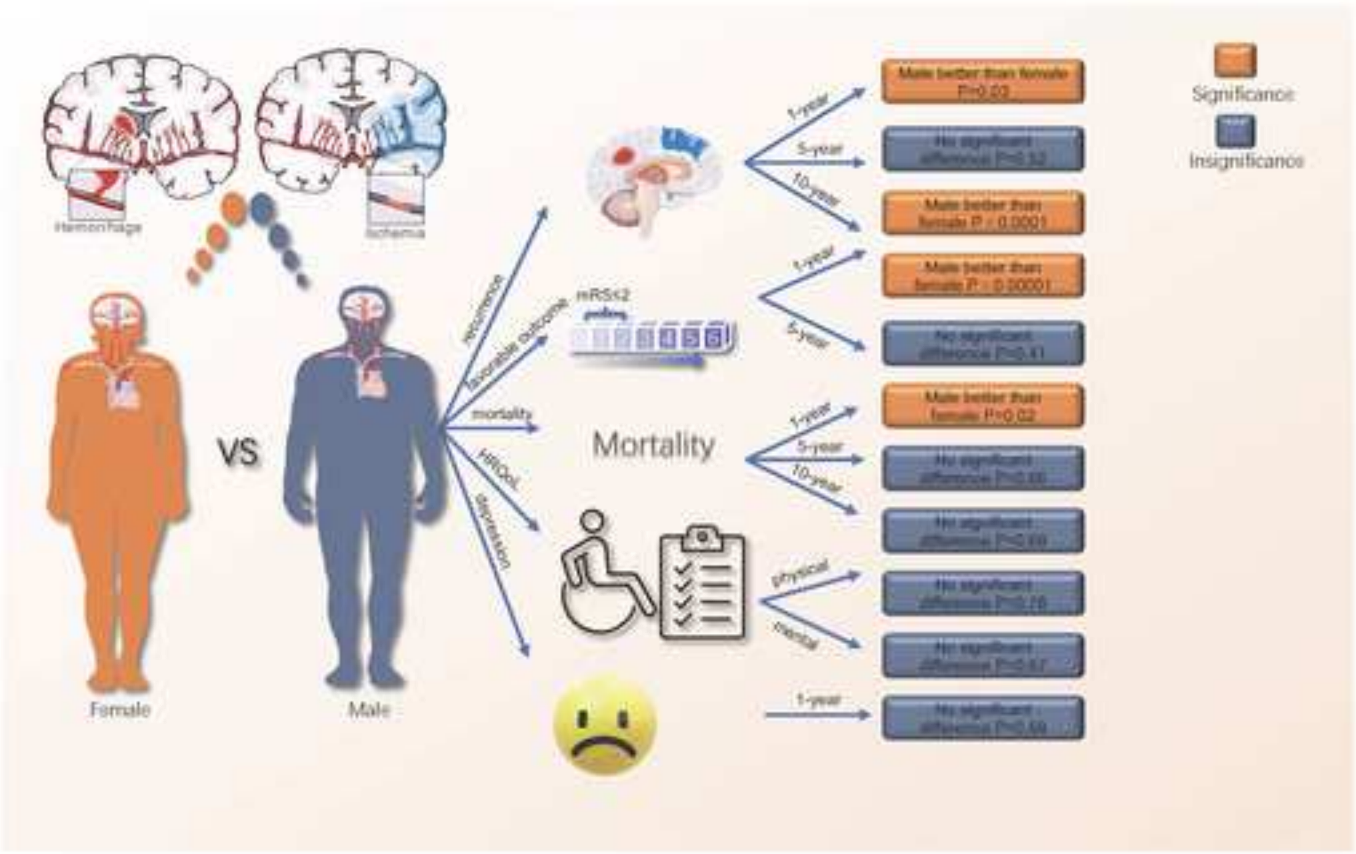

Click here to access/download
Supporting Information
supplement matierial for stroke outcome.pdf

PRISMA checklist

Click here to access/download
Supporting Information
PRISMA_2020_checklist.docx
