# Supplementary material for: Sex-based differences in long-term outcomes after stroke: A meta-analysis
Source: PLoS One. 2023 Apr 27;18(4):e0283204. doi: 10.1371/journal.pone.0283204 (PMC10138847; doi:10.1371/journal.pone.0283204)
Supplement: Attachment — Submitted filename: renamed_47dc1.docx [file pone.0283204.s004.docx]

February 14, 2023

Ahmed Nasreldein, MD

Academic Editor

**PLOS ONE**

Dear Editor Ahmed Nasreldein

Thank you so much for your e-mail dated 31 January 2022, providing yours and the Reviewers’ comments on our submission (Manuscript ID: PONE-D-23-00740) entitled “Sex-based differences in long-term outcomes after stroke: a meta-analysis”. Following your suggestions and the reviewers` comments, our manuscript has been revised completely and re-submitted, along with the point-by-point responses to the comments.

In the re-submitted manuscript, the revised parts have been marked in yellow. We believe that the revised manuscript can meet the publication standards of your journal.

Thank you again for your help in improving our manuscript.

Yours sincerely,

Feng Zheng

Professor and Director of Department of Neurosurgery,

The Second Affiliated Hospital,

Fujian Medical University

34 Zhongshan North Road

Quanzhou 362000

Fujian Province

P.R. of China

Tel: + (86)13225985688

Email: dr.feng.zheng@gmail.com

**Reviewer 1 evaluation: The authors address the important topic of gender differences in long-term outcome after stroke. They identified twenty-two cohort studies and analysed 84538 patients, of whom with 49.8% were women. Their results showed that women had a higher mortality after 1 and 5 years, higher rate of recurrent strokes and less favourable 1-year outcome. The results are very important and strengthen the need for further investigations in this area of stroke medicine. The results are a basis for the development of interventional studies aiming at gender equity**.

*Q1: The analysis is seems well conducted however, I cannot fully review the manuscript. I was not able to read Figure 1, which shows the main results. The image quality is too poor and the figure appears too blurry. Nothing can be identified. Same for Figure 4. It was not possible to review most of the results.*

**Response to the comment:** Thank you so much for your positive and constructive suggestions. Following your comments, the clear figures with better quality have been resubmitted. Please see the revised Figure 1, 2, 3, 4 below.

***Figure 1 Flow chart showing the search strategy***

***Figure 2 Forest plots showing primary outcomes between males and females. M-H Mantel-Haenszel statistic***

***Figure 3 Forest plot showing secondary outcomes between males and females. M-H: Mantel-Haenszel statistic***

***Figure 4 The comparison of long-term outcomes between males and females***

*Q2: I have the following comments to the introduction and methods:*

*Introduction: I suggest to omit the "general" sentence that stroke is a leading cause of death and disability. This Is well known and it would be much more attracting for readers to start with the topic, e.g. start with sentence two.*

**Response to the comments:** Thank you so much for your suggestion. Following your idea, the "general" sentence that stroke is a leading cause of death and disability has been omitted, and the revised manuscript is started with sentence two. Please see line 67-68.

***(Line 67-68, Introduction)***

Stroke affects female and male patients differently, although the reasons and mechanisms of these differences are unclear (1, 2).

*Q3: The authors describe that they used the "Cochrane risk bias assessment tool was used to assess the bias in randomized controlled studies". Was this the "Cochrane risk-of-bias tool for randomized trials (RoB 2)" tool? If so, it would be easier for the reader if this was indicated in the sentence in detail.*

*Just out of curiosity, what was the reason to not use the ROBINS-I tool for observational studies?*

**Response to the comments:** Yes, the "Cochrane risk bias assessment tool" in our manuscript was the "Cochrane risk-of-bias tool for randomized trials (RoB 2)" you mentioned, which has been replaced accordingly. Please see line 137-138.

Additionally, at the recommendation of *Handbook for Systematic Reviews of Interventions,* the Newcastle–Ottawa Scale (NOS) was adopted in this meta-analysis, which is the most popular tool for evaluating risk bias of observational cohort studies when performing meta-analysis. ROBINS-I is a new tool for evaluating risk bias in estimates of the effectiveness or safety (benefit or harm) of an intervention from studies that do not use randomized to allocate interventions. If possible, we would use this tool in our next studies.

***(Line 137-138, Methods)***

Cochrane risk-of-bias tool for randomized trials (RoB 2) was used to assess the bias in randomized controlled studies (17).

*Q4: From the text, I do not understand whether the sensitivity analysis for mortality was performed for 1 year and 5 years? On Page 10, lines 200-203 only one results is presented.*

**Response to the comments:** We appreciate your comment. Yes, sensitive analysis was performed for 1 year and 5 years. Due to no exact heterogeneity source detected after sensitivity analysis for the outcome of 1 year mortality, we just presented the results of sensitivity analysis for 5-year mortality. Following your idea, this issue has been corrected in the revised manuscript. Please see line 194-196 and line 254-256.

***(Line 194-196, Results)***

Due to the substantial heterogeneity in the assessments of above two outcomes (I^2^=90%, P<0.00001 and I^2^=92%, P<0.00001), a sensitivity analaysis was performed, without having detected the exact heterogeneity source.

***(Line 254-256, Discussion)***

In our meta-anlaysis, the conclusion that female patients with stroke had significantly higher 1-year mortality rates should be interpreted with caution because of the significant heterogeneity.

*Q5: In the paragraph addressing the secondary outcomes depression and health related quality of live, the authors describe that there was no significant difference between men and women. This is also discussed on page 12. Considering the serious heterogeneity of the studies with I2 between 68 and 97%, the conclusion should be that there is not enough evidence to answer this question.*

**Response to the comments:** Thank you for your constructive suggestion. There was no significant difference between men and women, and we have revised this conclusion accordingly. Please see line 277-279.

***(Line 277-279, Discussion)***

In our meta-analysis, the finding that no significant difference was found in depression and health-related quality of life between male and female, should be interpreted with cautioun because of the significant heterogeneity.

*Q6: Is any analysis available comparing data from men and women of the same age-group and disease severity?*

**Response to the comments:** Thank you so much for your constructive comments. Due to the lack of patient level data comparing long-term stroke outcomes between male and female, further analysis cannot be achieved in the present analysis, which has been addressed in the limitation of the revised manuscript. Please see line 294-296 below.

***(Line 294-296, Discussion)***

Furthermore, due to the lack of patient level data, further analysis including different age-groups and disease severity could not be performed in the present meta-analysis to identify long-term outcomes between male and female. Future studies are needed to address this issue.

**Reviewer 2 evaluation: I congratulate you on a well-written article which attempts to highlight an important yet less addressed issue in stroke. I would like to however point out few discrepancies and urge you to correct these.**

*Q1: While the results clearly state that the mortality rates were higher for women at 1 year and 10 years, repeatedly in the paper (abstract, body and conclusion) it is mentioned at 1 and 5 year significant mortality rates. I believe this is a mistake and needs to be edited.*

**Response to the comments:** Thank you for your constructive and positive comments. Yes, this is a mistake, which has been corrected accordingly. Please see line 58, line 301 below.

***(Line 58, Abstract)***

In this meta-analysis, the 1- and 10-year mortality and stroke recurrence rates were higher in female patients than in male patients after stroke.

***(Line 301, Conclusion)***

This meta-analysis found that 1- and 10-year mortality, as well as stroke recurrence rates, were higher in female patients than in male patients after stroke.

*Q2: Again in the results HRQoL shows no difference between males and females, however in the discussion authors have highlighted and discussed a difference in the poorer mental and physical QOL in women.*

**Response to the comments:** Thank you for your comments. Yes, there is no significant difference in the outcomes of mental and physical QOL between male and female. The issue mentioned has been revised accordingly. Please see line 277-279 below.

***(Line 277-279, Discussion)***

In our meta-analysis, the finding that no significant difference was found in depression and health-related quality of life between male and female, should be interpreted with cautioun because of the significant heterogeneity.

*Q3: The consort figure also has discrepancies. The number of excluded articles with reasons do not tally together and some information is either missing or incorrect.*

**Response to the comments:** Thank you for your constructive comments. Following your suggestions, the number of excluded articles with reasons, along with the missing or incorrect information, has been revised. Please see line 174-178, Figure 1 below.

***(Line 174-178, Results)***

The full text of the remaining 227 articles was assessed for eligibility, resulting in the exclusion of 150 studies with outcomes not stratified by sex and 37 conference abstracts without a complete text. Three studies were excluded because their data were not extractable. Fifteen of the remaining articles were excluded due to a lack of long-term outcome data based on sex-based differences.

***(Figure 1 Flow chart showing the search strategy)***
